# EHMTI-0146. Topiramate in migraine prevention

**DOI:** 10.1186/1129-2377-15-S1-G32

**Published:** 2014-09-18

**Authors:** S Ristic, D Ristic

**Affiliations:** 1 Department of Neurology, Epilepsy Unit, Nis, Serbia; 2 Institutae of Pulmonary Disaeses, Institutae of Pulmonary Disaeses, Nis, Serbia

## Purpose

The present analysis of pooled data from those 3 trials was performed to characterize the efficacy and adverse events of topiramate for migraine prevention in subjects who had used other migraine preventive medications.

## Metods

We were analyzed patients with migraine, who had used migraine preventive medications, within approximately 8 months period.Patients were admitted to the Department of Neurology in Nis, after observing their miagraine atack, during the period from January-December 2013.All of the patients have been diagnostically examend by:interictal EEG, head CT, MRI,MRA angiography.We analyzed monthly migraine frequency from baseline period to endpoint. We compared different dosage of topiramate, so as the presence od adverse events.We started with 25mg/day od topiramate and this lasted for 7 days and we incresed dosage of topiramate 25 mg/week.Maximum dosage was 200 mg/day.

## Results

Of sum of 167 patients, 96 (57,48%)female, aged between 21-59 years (mean age 39,2) were recruited. Subjects were treated with topiramate(50, 100 or 200 mg/day). More subjects on topiramate 50 mg./day (41%), 100 mg./day (63%) and 200 mg./day (54%) exhibited ≥ 50% reductions in monthly migraine frequency. Most common adverse event was paresthesis, incidence was 11%, fatigue 3%, nausea 2%. Mean duration of paresthesia was 19 days. Cognitive sympthoms was registered only in 2 patients.

## Conclusion

In subjects who had previously taken other migraine preventives, treatment with topiramate100 mg/day and 200 mg/day significantly reduced mean monthly migraine frequency. In our study, the lower dose of topiramate exhibited similar efficacy. Adverse events is rarely and most frequent is paresthesia which disappeares in 3 weeks.

No conflict of interest.

